# Population prevalence of myopia, glasses wear and free glasses acceptance among minority versus Han schoolchildren in China

**DOI:** 10.1371/journal.pone.0215660

**Published:** 2019-04-18

**Authors:** Min Hu, Yuan Zhou, Shanshan Huang, Nathan Congdon, Ling Jin, Xiuqin Wang, Ruth Hogg, Hong Zhang, Yongkang Cun, Luhua Yang, Xianshun Li, Chaoguang Liang

**Affiliations:** 1 The Second People’s Hospital of Yunnan, Kunming, China; 2 Centre for Public Health, Queen’s University Belfast, Belfast, Ireland, United Kingdom; 3 State Key Laboratory of Ophthalmology, Zhongshan Ophthalmic Center, Guangzhou, China; 4 Orbis International, New York City, New York, United States of America; 5 Affiliated Hospital of Guangdong Medical University, Zhanjiang, Guangdong, China; 6 Honghe First People’s Hospital, Honghe, Yunnan, China; 7 Dehong People’s Hospital, Dehong, Yunnan, China; 8 Jianchuan People’s Hospital, Jianchuan, Yunnan, China; 9 Chuxiong People’s Hospital, Chuxiong, Yunnan, China; 10 Lancang County First People’s Hospital, Lancang, Yunnan, China; Faculty of Medicine, Cairo University, EGYPT

## Abstract

**Aim:**

To measure myopia, glasses wear and free glasses acceptance among minority and Han children in China.

**Methods:**

Visual acuity testing and questionnaires assessing ethnicity, study time, and parental and teacher factors were administered to a population-based sample of 9–12 year old minority and Han children in Yunnan and Guangdong, and their teachers and parents. Refraction was performed on children with uncorrected visual acuity (VA) < = 6/12 in either eye, and acceptance of free glasses assessed.

**Main outcome measures:**

Baseline myopia (uncorrected visual acuity < = 6/12 in > = 1 eye and spherical equivalent refractive power < = -0.5D in both eyes); baseline glasses wear; free glasses acceptance.

**Results:**

Among 10,037 children (mean age 10.6 years, 52.3% boys), 800 (8.0%) were myopic, 4.04% among Yunnan Minority children (OR 0.47, 95%CI 0.33, 0.67, P<0.001), 6.48% in Yunnan Han (OR 0.65, 95%CI 0.45, 0.93, P = 0.019), 9.87% in Guangdong Han (Reference). Differences remained significant after adjusting for study time and parental glasses wear. Difference in baseline glasses ownership (Yunnan Minority 4.95%, Yunnan Han 6.15%, Guangdong Han 15.3%) was not significant after adjustment for VA. Yunnan minority children (71.0%) were more likely than Yunnan Han (59.6%) or Guangdong Han (36.8%) to accept free glasses. The difference was significant after adjustment only compared to Guangdong Han (OR 3.34, 95% CI 1.62, 6.90, P = 0.001).

**Conclusion:**

Myopia is more common among Han children and in wealthier Guangdong. Baseline differences in glasses wear could be explained by student, teacher and parental factors. Yunnan Minority children were more likely to accept free glasses.

## Introduction

Refractive error (RE) is a common eye disorder and the leading cause of visual impairment and blindness in children worldwide. [1] Myopia is the most common RE among school-aged children, and tends to increase with age and additional schooling. [2] China has among the highest prevalence of childhood myopia in the world. [3–9] Among the 13 million children with visual impairment due to uncorrected refractive error, nearly half live in China. [1]

Wearing glass is a safe and effective treatment for myopia, but evidence suggests that only 15–20% of rural and urban migrant children in China who need glasses have them. [10, 11] Most of this evidence, however, is drawn from children of Han ethnicity, a group comprising more than 90% of China’s population. Little information exists, however, about rates of myopia and spectacle wear among China’s ethnic minorities,[12] over 50 separate groups who together comprise some 100 million people. Their different cultures, lifestyle and genes could be associated with very different rates of myopia than observed among the Han.

We carried out a randomized trial on spectacle acceptance and wear among a random sample of primary school children in Guangdong and Yunnan provinces. [13] A high proportion of children in the Yunnan sample came from a variety of different minority groups. The purpose of the present study is to explore whether there are differences in the prevalence of myopia, glasses use or the acceptance of free glasses between minority and Han Chinese school children, and to better understand factors contributing to these differences.

## Methods

This study was approved by the Institutional Review Boards at Stanford University (Palo Alto, USA), the Zhongshan Ophthalmic Center (Guangzhou, China) and Yunnan Red Cross Hospital (Kunming, China). Permission was received from local boards of education in each region and the principals of all schools, and written informed consent was provided by at least one parent on behalf of all children. The principles of the Declaration of Helsinki were followed throughout.

The study was conducted in Guangdong and Yunnan provinces, China. Guangdong is one of China’s richest provinces, with a per capita gross domestic product (GDP) in 2015 of US$10,838, 8th among China’s 31 administrative divisions. [14] The population is 97.1% Han. [15] Yunnan (2015 GDP of US$4658) ranks second from bottom of China’s administrative divisions in wealth, [14] and 38% of its population are minorities.[16]

The study methods are described elsewhere in detail, and are summarized here for reference. [13] From a list of all 601 primary schools (362 in Guangdong and 239 in Yunnan) in 9 randomly-selected counties in Guangdong and Yunnan, we randomly selected 138 (88 schools in Guangdong and 50 in Yunnan), with the number of schools selected in each county being determined by population size. Within each sampled school, we randomly selected one class in each of the fourth and fifth grades (likely age range 9–12 years), if there was more than one class per grade level.

### Visual acuity assessment

Visual acuity screening was conducted at schools in well-lit rooms during daylight hours. Children’s visual acuity was tested in the right and then the left eye by two trained volunteer screeners using Early Treatment Diabetic Retinopathy Study[17] tumbling E charts (Precision Vision, La Salle, IL) at a distance of 4m. Acuity was measured with and without habitually-worn correction for those children owning glasses, with children having been reminded to bring their glasses in advance of the examination. If children correctly identified at least 4 or 5 optotypes on the top line (6/60), they were re-examined at 6/30, 6/15 and then line by line to 6/3. We defined visual acuity for an eye as the lowest line on which four of five optotypes were read correctly. If the top line could not be read at 4m, the participant was tested at 1m and the measured visual acuity was divided by four.

### Refraction

All children with uncorrected visual acuity < = 6/12 in either eye underwent cycloplegia with up to three drops of cyclopentolate 1% in each eye after anesthesia with topical proparacaine hydrochloride 0.5%. Children then underwent automated refraction (Topcon KR 8900, Tokyo, Japan) with subjective refinement by an experienced refractionist. Children of parents refusing permission for cycloplegia (274/882 = 31.1%) underwent subjective refinement of the non-cycopleged value from the auto-refractor by an experienced refractionist in each eye using a target at four meters distance.

### Questionnaires

At baseline (September 2014), enumerators administered questionnaires to children (S1 Table), including questions on race (Han versus various minority groups), age, sex, glasses wear, awareness of refractive status, belief that wearing glasses harms children’s vision, parental living condition and education, and ownership of a list of 16 selected items as an index of family wealth (the Family Affluence Scale II, previously validated among adolescents in China. [18]) At endline (June 2015), questionnaires were administered on glasses ownership, glasses wear, parental attitude toward wearing glasses and subjective evaluation of project glasses.

### Provision of free glasses

As part of the parent trial, in October 2014, children were randomized by school to receive either a glasses prescription and letter to the parents informing them of the refractive status of their child; a voucher exchangeable for free glasses at the local county hospital; or vouchers for free glasses plus the offer of “upgrade glasses” (with scratch-proof lenses and popular designs). County hospitals were located at a median distance of 27 km (Guangdong: Range 3–63 km; Yunnan: Range 4–113 km) from the children’s township of residence.

### Outcome assessment

Myopia was defined as having uncorrected visual acuity < = 6/12 in at least one eye and spherical equivalent refractive error < = -0.5 D in both eyes. Needing glasses was defined as having uncorrected visual acuity < = 6/12, correctable to > 6/12 in either eye, together with refractive power in both eyes in a range previously demonstrated[19] to be associated with significantly greater improvement with visual acuity when corrected (myopia < = -0.5D, hyperopia > = +2.0 D, or astigmatism > = 0.75 D). Glasses wear at baseline was defined as having glasses at school, having been told before to bring them. Acceptance of the offered free glasses (among those children randomized to receive vouchers) was based on records maintained by the county hospitals.

### Statistical methods

Baseline characteristics of children by province and ethnicity were presented as mean (SD, standard deviation) for continuous data with normal distribution, and frequency (percentage) for categorical data. We calculated family wealth by summing the value, as reported in the China Rural Household Survey Yearbook (Department of Rural Surveys, National Bureau of Statistics of China, 2013), of items on the list of 16 owned by the family. [18] Refractive power was defined throughout as the spherical equivalent: the spherical power plus half the cylindrical power.

The comparison of baseline characteristics between Minority and Han children was done using linear regression for continuous variables, logistic regression for binary variables and ordinal logistic regression for ordinal categorical variables, adjusting in all cases for clustering effects within schools. Logistic regression was used to assess the impact of factors associated with myopia, baseline spectacle ownership and acceptance of free glasses. Children in the Control Group, who were not offered free glasses, were excluded from the analysis on acceptance of free spectacles. All variables significant at the p< = 0.2 level in the simple regression models were included in the multiple regression model. Clustering effect within schools was taken into account in all regression analyses. All statistical analyses were done using a commercially available software package (Stata 13.1, StataCorp, College Station TX, USA).

## Results

Among 10,234 students in the selected classes that completed baseline questionnaires and vision screening, 165 (1.61%) minority children from Guangdong and 32 (0.31%) children with missing ethnicity data were excluded from analysis, leaving 10,037 children (98.1%): 6293 Guangdong Han (62.7%; mean age 10.6 years, 53.6% males), 1142 Yunnan Han (11.4%, mean age 10.5 years, 52.5% males) and 2602 Yunnan minority (25.9%, mean age 10.6 years, 49.1% males). Among these, 9087 (90.5%) passed vision screening, 950 (9.46%) failed screening and 800 (7.97%) were myopic (uncorrected visual acuity < = 6/12 in at least one eye and SE < = -0.5 D in both eyes). These included 621 Guangdong Han (77.6%), 74 Yunnan Han (9.25%) and 105 Yunnan minority (13.1%) children. The prevalence of myopia was 9.87% among Guangdong Han children, 6.48% among Yunnan Han and 4.04% among Yunnan Minority. (Table 1).

**Table 1:** Ethnic composition and characteristics of children participating in a study of spectacle use in Guangdong and Yunnan. China (N = 10,037).

Ethnicity	n (%)	Age (year) Mean (SD)	Male sex n (%)	Myopia n (%)†
**Guangdong Han**	6293 (62.7)	10.6 (0.96)	3375 (53.6)	621 (9.87)
**Yunnan Han**	1142 (11.4)	10.5 (0.91)	600 (52.5)	74 (6.48)
**Yunnan Minority**	2602 (25.9)	10.6(0.99)	1277(49.1)	105(4.04)
Dai	176 (6.76)	10.1 (0.91)	85 (48.3)	12 (6.82)
Yi	542 (20.8)	10.7 (1.03)	257 (47.4)	28 (5.17)
Bai	423 (16.3)	10.5 (0.75)	227 (53.7)	27 (6.38)
Lahu	549 (21.1)	10.5 (0.97)	294 (53.6)	9 (1.64)
Jingpo	145 (5.57)	10.7 (0.77)	69 (47.6)	5 (3.45)
Wa	206 (7.92)	10.4 (1.06)	81 (39.3)	3 (1.46)
Hani	203 (7.80)	10.4 (1.00)	103 (50.7)	7 (3.45)
Miao	183 (7.03)	11.1 (1.19)	83 (45.4)	4 (2.19)
Zhuang	45 (1.73)	10.7 (0.87)	23 (51.1)	5 (11.1)
De’ang	78 (3.00)	10.7 (1.00)	30 (38.5)	0 (0.00)
Other	52 (2.00)	10.7 (1.12)	25 (48.1)	5 (9.62)
Total	2602 (25.9)	10.6 (0.99)	1277 (49.1)	105 (4.04)

^†^ Myopia was defined as uncorrected visual acuity < = 6/12 in at least one eye and spherical equivalent < = -0.5 Diopters in both eyes.

At baseline, Yunnan minority children spent less time studying (P<0.01) and had less prosperous families (P<0.001) than Yunnan Han children, but did not differ in parental out-migration for work, educational level and glasses ownership. (Table 2) Comparing Yunnan minority and Guangdong Han children, Yunnan minority children were significantly more likely to be the only child in the family (17.5 versus 10.6%, p<0.001); less likely to be male (49.1 versus 53.6%, P<0.001); and had shorter study hours (P<0.001) and less prosperous families (P<0.001). Their parents were less likely to have 12 years of education (12.8 versus 31.2%, p<0.001), wear glasses (4.04 versus 12.9%, P<0.001) and to have out-migrated for work (13.1 versus 23.0%, p<0.001). (Table 2).

**Table 2:** Comparison of baseline characteristics between minority and Han children (N = 10,037).

Characteristics	All (N = 10,037)	YN Minority (n = 2602) (1)	YN Han (n = 1142) (2)	GD Han (n = 6293) (3)	P-value (1) versus (2)	P-value (1) versus (3)	Missing data
Age, years, Mean (SD)*	10.6 (0.96)	10.6 (0.99)	10.5 (0.91)	10.6 (0.96)	0.207	0.710	4
**Male sex, n (%)**†	5252 (52.3)	1277 (49.1)	600 (52.5)	3375 (53.6)	0.057	**<0.001**	0
**Myopia, n (%)**	800 (7.97)	105(4.04)	74 (6.48)	621 (9.87)	**0.010**	**<0.001**	0
**Only child in family, n (%)**†	1288 (12.8)	455 (17.5)	168 (14.7)	665 (10.6)	0.140	**<0.001**	0
**At least one parent with > 12 years education, n (%)**†	2456 (24.5)	333 (12.8)	162 (14.2)	1961 (31.2)	0.555	**<0.001**	0
**Both parents away from the home the majority of time, n (%)**†	1973 (19.7)	341 (13.1)	184 (16.1)	1448 (23.0)	0.127	**<0.001**	0
**At least one parent wears glasses, n (%)**†	972 (9.68)	105 (4.04)	55 (4.82)	812 (12.9)	0.266	**<0.001**	0
**Study time each day after school (hours)** ‡							9
<0.5 hr	4704 (46.9)	1480 (56.9)	520 (45.5)	2704 (43.0)	**0.010**	**<0.001**	
0.5–1 hr	3191 (31.8)	686 (26.4)	426 (37.3)	2079 (33.1)			
>1 hr	2133 (21.3)	436 (16.8)	196 (17.2)	1501 (23.9)			
**Family wealth**‡							83
Bottom tercile	3287 (33.0)	1439 (55.6)	506 (44.5)	1342 (21.5)	**<0.001**	**<0.001**	
Middle tercile	3322 (33.4)	535 (20.7)	253 (22.3)	2534 (40.7)			
Top tercile	3345 (33.6)	613 (23.7)	378 (33.3)	2354 (37.8)			
**Blackboard use**‡							13
Less than half	5404 (53.9)	1391 (53.5)	608 (53.3)	3405 (54.2)	0.855	0.681	
Half of teaching	2775 (27.7)	809 (31.1)	344 (30.2)	1622 (25.8)			
More than half	1845 (18.4)	401 (15.4)	189 (16.6)	1255 (20.0)			

SD = standard deviation

* Linear regression was used for the comparison adjusting cluster effects within school.

^†^ Logistic regression was used for the comparison adjusting cluster effects within school.

^‡^ Ordinal logistic regression was used for the comparison adjusting cluster effects within school.

Among 768 children needing glasses, 4.95% (5/101) Yunnan minority, 6.15% (4/65) Yunnan Han and 15.3% (92/602) Guangdong Han children owned them at baseline. Among children with uncorrected visual acuity in the better seeing eye < = 6/19 to > = 6/48, the rate of ownership was 21.7% (58/267) in Guangdong Han children, significantly higher than the Yunnan minority (2/41 = 4.88%, P = 0.010). (Table 3).

**Table 3:** Prevalence of glasses ownership and free glasses acceptance among minority and Han children needing glasses (myopia < = -0.5D or hyperopia > = +2.0 D or astigmatism > = 0.75 D in both eyes and uncorrected visual acuity < = 6/12, correctable to > 6/12 in either eye) (N = 768).

	Yunnan Minority (n = 101)		Yunnan Han (n = 65)		Guangdong Han (n = 602)	
	Owned glasses at baseline†	Accepting free Study Glasses§	Owned glasses at baseline†	Accepting free Study Glasses§	Owned glasses at baseline†	Accepting free Study Glasses§
**All children needing glasses**	5/101 (4.95)	49/69 (71.0)	4/65 (6.15)	28/47 (59.6)	92/602 (15.3)**	158/429 (36.8)***
**Myopia in better seeing eye**‡						
-0.5D to -1.5D	2/36 (5.56)	14/24 (58.3)	0/22 (0.00)	12/15 (80.0)	3/172 (1.74)	50/133 (37.6)
-1.5D to -2.5D	0/41 (0.00)	25/27 (92.6)	0/25 (0.00)	8/18 (44.4)**	17/212 (8.02)	57/150 (38.0)***
-2.5D to -3.5D	1/13 (7.69)	5/9 (55.6)	2/10 (20.0)	4/8 (50.0)	32/124 (25.8)	26/78 (33.3)
-3.5D to -4.5D	0/6 (0.00)	4/6 (66.7)	1/4 (25.0)	2/3 (66.7)	20/46 (43.5)	9/29 (31.0)
< = -4.5D	2/4 (50.0)	1/3 (33.3)	1/3 (33.3)	2/2 (100.0)	14/25 (56.0)	10/20 (50.0)
Total	5/100 (5.00)	46/69 (71.0)	4/64 (6.25)	28/46 (60.9)	86/579 (14.9)**	152/410 (37.1)
**Hyperopia in better seeing eye (> = +2.0D)** ‡	0/1 (0.00)	0/0 (0.00)	0/0 (0.00)	0/0 (0.00)	4/8 (50.0)	2/7 (28.6)
**Astigmatism in both eyes (> = 0.75 D)**	1/2 (50.0)	0/0 (0.00)	0/4 (0.00)	1/3 (33.3)	22/76 (29.0)	24/55 (43.6)
**Uncorrected visual acuity in better seeing eye** ‡						
< = 6/12 to > = 6/18	3/38 (7.89)	18/23 (78.3)	1/26 (3.85)	11/20 (55.0)	12/203 (5.91)	52/151 (34.4)
< = 6/19 to > = 6/48	2/41 (4.88)	21/30 (70.0)	3/26 (11.5)	13/20 (65.0)	58/267 (21.7)*	66/177 (37.3)
< 6/48	0/4 (0.00)	4/4 (100.0)	0/2 (0.00)	1/1 (100.0)	14/27 (51.9)	8/20 (40.0)

^§^ Children in control group were excluded.

^†^ Brought glasses to school after being instructed to the previous day

^‡^Better-seeing eye was the eye with better uncorrected visual acuity at baseline.

*P-value<0.05,

**P-value<0.01,

***P-value<0.001.

Logistic regression was used for the comparisons of Yunnan Minority vs. Yunnan Han and Yunnan Minority vs. Guangdong Han among each of groups with adjusting for cluster effects within school.

Among children needing glasses, 545 (71.0%) were randomly selected by school to receive free glasses (429 Guangdong Han [78.7%], 47 Yunnan Han [8.62%] and 69 Yunnan minority [12.7%].) Among selected children, 71% (49/69) of Yunnan minority and 59.6% (28/47) of Yunnan Han children claimed their free glasses at participating hospitals, significantly greater than for Guangdong Han children (158/429 = 36.8%, p<0.001). Among children with myopia -1.5D to -2.5D, the free glasses acceptance rate among Yunnan minority children was significantly higher than for Yunnan Han (25/27 = 92.6% versus 8/18 = 44.4%, P = 0.003) and Guangdong Han children (57/150 = 38.0%, P<0.001). (Table 3).

Minority children had significantly lower risk of myopia than Han children in both univariate and multivariate models (Table 4) Besides ethnicity, other factors significantly associated with myopia in multivariate models included: male sex (OR 0.70, 95%CI 0.60, 0.81, P<0.001); at least one parent wearing glasses (OR 2.18, 95%CI 1.81, 2.63, P<0.001; studying more than one hour daily after school (OR 1.41, 95%CI 1.19, 1.66, P<0.001); family wealth in the top tercile (OR 1.46, 95%CI 1.18, 1.82, P<0.001); and both parents out-migrated for work (OR 0.88, 95%CI 0.79, 0.97, P = 0.011). (Table 4).

**Table 4:** Logistic regression model of factors potentially associated with myopia among minority and Han children adjusting for cluster effects within school (N = 10,037).

Variable	Simple Logistic Regression Analysis			Multiple Logistic Regression Analysis*(N = 9,938)		
	Odds ratio	95% confidence interval	P-value	Odds ratio	95% confidence interval	P-value
**Ethnic group**						
Yunnan Minority vs. Yunnan Han	0.61	(0.89, 0.42)	**0.010**	0.65	(0.45, 0.93)	**0.019**
Yunnan Minority vs. Guangdong Han	0.38	(0.27, 0.55)	**<0.001**	0.47	(0.33, 0.67)	**<0.001**
**Age (Years)**	1.02	(0.94, 1.10)	0.666			
**Male sex**	0.72	(0.62, 0.83)	**<0.001**	0.70	(0.60, 0.81)	**<0.001**
**Only child in family**	1.10	(0.87, 1.40)	0.416			
**At least one parent with > 12 years education**	1.35	(1.12, 1.63)	**0.002**	1.05	(0.87, 1.25)	0.625
**Both parents away from the home the majority of time**	0.90	(0.82, 1.00)	0.056	0.88	(0.79, 0.97)	**0.011**
**At least one parent wears glasses**	2.59	(2.15, 3.12)	**<0.001**	2.18	(1.81, 2.63)	**<0.001**
**Study time each day after school (hour)**						
<0.5	Reference			Reference		
0.5–1	1.30	(1.10, 1.53)	**0.002**	1.17	(1.00, 1.37)	**0.049**
>1	1.67	(1.38, 2.02)	**<0.001**	1.41	(1.19, 1.66)	**<0.001**
**Family wealth (Bottom tercile as reference)**						
Middle tercile	1.79	(1.41, 2.26)	**<0.001**	1.35	(1.07, 1.69)	**0.010**
Top tercile	1.92	(1.53, 2.43)	**<0.001**	1.46	(1.18, 1.82)	**<0.001**
**Blackboard use (Less than half as reference)**						
Half of teaching	0.87	(0.70, 1.09)	0.234	0.89	(0.73, 1.10)	0.284
More than half	0.85	(0.66, 1.09)	0.195	0.91	(0.72, 1.16)	0.435

* Variables in simple regression at p< = 0.2 were included in the multiple regression.

There was no difference in baseline glasses ownership between Yunnan minority and Yunnan Han children (p = 0.719). Though unadjusted odds of spectacle ownership were significantly lower for Yunnan minority than Guangdong Han children (OR 0.29, 95%CI 0.12, 0.68, P = 0.004), this became non-significant with adjustment for other determinants of wear (Table 5). Factors significantly associated with baseline glasses ownership in multivariate models included: uncorrected VA<6/18 in both eyes (OR 7.34, 95%CI 4.19, 12.9, P<0.001); at least one parent wearing glasses (OR 1.68, 95%CI 1.04, 2.74, P = 0.036); studying 30 minutes to one hour after school each day (less than half hour as reference: OR 1.91, 95%CI 1.15, 3.17, P = 0.013); and teachers’ support for students wearing glasses in class (OR 4.20, 95%CI 1.03, 17.1, P = 0.046) and advising them to purchase glasses (OR 3.12, 95%CI, 1.04, 9.32, P = 0.042). (Table 5).

**Table 5:** Logistic regression model of factors potentially affecting baseline spectacle ownership and acceptance of free glasses among myopic minority and Han children needing glasses adjusting for cluster effects within school.

Variable	Baseline Spectacle Ownership (N = 768)			Acceptance of Free Glasses (N = 545)§		
	Simple regression	Multiple regression† (N = 749)		Simple regression	Multiple regression† (N = 529)	
	OR (95% CI)	OR (95% CI)	P-value	OR (95% CI)	OR (95% CI)	P-value
**Student factors**						
**Ethnic group**						
Yunnan Minority vs. Yunnan Han	0.79 (0.23, 2.79)	0.84 (0.22, 3.19)	0.797	1.66 (0.58, 4.78)	2.06 (0.65, 6.44)	0.215
Yunnan Minority vs. Guangdong Han	0.29 (0.12, 0.68)**	0.39 (0.14, 1.08)	0.069	4.20 (2.05, 8.62)***	**3.34 (1.62, 6.90)**	**0.001**
**Age (Years)**	0.85 (0.69, 1.05)‡	1.09 (0.67, 1.77)	0.737	0.99 (0.83, 1.19)		
**Male sex**	1.47 (0.92, 2.34) ‡	1.58 (0.97, 2.55)	0.065	0.86 (0.62, 1.20)		
**Wearing glasses at baseline**	**/**	**/**	**/**	1.05 (0.60, 1.84)		
**Uncorrected VA < 6/18 in both eyes**	7.05 (4.07, 12.2)***	**7.34 (4.19, 12.9)**	**<0.001**	0.89 (0.62, 1.30)		
**At least one parent wears glasses**	2.22 (1.44, 3.44)***	**1.68 (1.04, 2.74)**	**0.036**	0.91 (0.58, 1.42)		
**Both parents away from the home the majority of time**	0.82 (0.60, 1.15)			0.82 (0.64, 1.05) ‡	0.84 (0.65, 1.09)	0.193
**Only child in family**	1.52 (0.98, 2.38) ‡	1.00 (0.53, 1.92)	0.990	1.14 (0.70, 1.86)		
**At least one parent with > 12 years education**	1.65 (1.17, 2.31)**	1.51 (0.97, 2.35)	0.071	0.77 (0.50, 1.17)		
**Study time each day after school (hour)**						
<0.5	Reference	Reference		Reference	Reference	
0.5–1	1.80 (1.14, 2.84)*	**1.91 (1.15, 3.17)**	**0.013**	0.95 (0.62, 1.45)	0.98 (0.64, 1.50)	0.922
>1	2.10 (1.19, 3.73)*	1.76 (0.92, 4.24)	0.088	0.56 (0.36, 0.87)*	**0.57 (0.36, 0.89)**	**0.014**
**Family wealth (Bottom tercile as reference)**						
Middle tercile	2.45 (1.11, 5.40)*	2.07 (0.85, 5.04)	0.110	0.83 (0.48, 1.45) ‡	1.20 (0.65, 2.22)	0.555
Top tercile	2.66 (1.33, 5.31)**	1.98 (0.92, 4.24)	0.081	0.60 (0.36, 0.99)*	0.74 (0.42, 1.31)	0.299
**Blackboard use (Less than half as reference)**						
Half of teaching	1.40 (0.73, 2.67)			1.34 (0.69, 2.59)		
More than half	1.29 (0.69, 2.43)			1.09 (0.61, 1.93)		
**Teacher factors**						
**Teacher’s age above median (38) (median or below as reference)**	0.92 (0.58, 1.46)			1.78 (1.09, 2.90)*	**1.67 (1.12, 2.50)**	**0.012**
**Female teacher**	1.12 (0.55, 2.28)			0.58 (0.34, 0.98)*	0.77 (0.46, 1.29)	0.322
**Teacher believes wearing glasses harms children’s vision (Disagree as reference)**	0.73 (0.50, 1.07) ‡	0.79 (0.56, 1.12)	0.184	1.32 (0.94, 1.85) ‡	1.25 (0.90, 1.73)	0.191
**Teacher support students wearing glasses in class**	3.84 (1.21, 12.1)*	**4.20 (1.03, 17.1)**	**0.046**	2.15 (0.74, 6.27) ‡	1.78 (0.81, 3.90)	0.149
**Teacher advised children to purchase glasses**	2.31 (1.32, 4.06)**	**3.12 (1.04, 9.32)**	**0.042**	6.78 (3.27, 14.1)***	**4.07 (2.19, 7.57)**	**<0.001**

^§^ The kids among control group were excluded from the regression analysis for the acceptance of free glasses.

^†^ Variables in simple regression at p< = 0.2 were included in the multiple regression.

* P-value<0.05,

** P-value<0.01,

*** P-value<0.001

^‡^ P-value>0.05 and < = 0.2

Yunnan minority children were significantly more likely to accept free glasses when compared with Guangdong Han children in multivariate models (OR 3.34, 95%CI 1.62, 6.90, P = 0.001), but their acceptance rates did not differ significantly when compared with Yunnan Han children (P = 0.345). Students who studied more than one hour after school (OR 0.57, 95%CI 0.36, 0.89, P = 0.014) were significant less likely to accept free glasses, while those with teachers above the median age (38 years) (OR 1.67, 95%CI 1.12, 2.50, P = 0.012) and who were advised by their teachers to purchase glasses (OR 4.07, 95%CI 2.19, 7.57, P<0.001) were significantly more likely to accept them. (Table 5).

## Discussion

The importance of the current study lies in the fact that very little population-based information exists about prevalence[12] and especially treatment of refractive error among ethnic minority children in China. The majority of Chinese people (92%) are from the Han group, but nearly 8% or 110 million Chinese are from 55 diverse minority ethnic groups. [20] Many minority groups live in sparsely populated, relatively impoverished areas, resulting in differences which are socioeconomic as well as cultural. [21, 22] Genetic differences between minority and Han groups have also been identified. [23, 24]

Evidence suggests that minority children and adults have worse health outcomes compared to Han, including infant mortality rates three times as high,[25] worse mental health status among ethnic minority college students,[26] lower breast cancer survival[27] and significantly higher rates of infection with human immunodeficiency virus (HIV) and Hepatitis C.[28]

In the current study, we found the prevalence of myopia among Han children to be significantly higher than for minority children. Although Han children had significantly longer study times and wealthier families compared with minority children, this could not entirely explain the differences in myopia prevalence. However, due to our lack of data on outdoor activity, we cannot rule out possibility that environment factors explain the difference in myopia prevalence between racial groups. Nonetheless, it is important for program planners to know that the myopia prevalence among minority children is only half that among the Han. This finding is consistent with a school based cross-sectional study carried out in Turpan, in which the lowest myopia prevalence was reported among the Uyghur minority (13%) and the highest among Han (27%), with the Hui minority intermediate (18%).^12^ A similar results was found in a Yunnan study by Yang et al, where the prevalence of myopia was 71.7% among Han and 35.7% for minority children.[29]

The main significance of our findings with regard to spectacle use was the very low rates, less than one in six, among all ethnic and geographic groups. A potential reason for glasses non-wear among myopic children in the current study may be associated with the widespread misunderstanding in China that young children wearing glasses might damage their visual acuity. [30, 31] The lower unadjusted rate of glasses use among Yunnan children may also relate to poor access to health services, which has been well documented there. [32, 33]

Given their low rates of observed spectacle use, it is very encouraging that minority Yunnan had the highest rates of acceptance of free glasses, with nearly three-quarters presenting to hospital in order to obtain spectacles. This result is consistent with previous studies showing that providing free glasses could significantly improve use among children in low-income rural areas. [34, 35] Our own work in under-served, rural Chinese populations has similarly suggested that uptake of free glasses approached 80%, and resulted in a doubling of rates of wear. [36] However, only about a third (36.8%) of families in wealthier Guangdong were willing to travel to receive free spectacles in the current study.

The strengths of this report include its population-based design, random selection of the sample, large size and inclusion of substantial numbers of both Han and minority children. Detailed information was collected not only on prevalence of myopia and spectacle wear, but also on the very important behavior of acceptance of free glasses. Limitations must also be acknowledged: We did not refract all children, but rather only those failing vision screening. This limits comparability of our figures on refractive error prevalence with other studies, but does not affect comparisons within the study between Han and minority children. Nearly a third of families refused cycloplegia on behalf of their children and had to undergo subjective refinement by an experienced optometrist as a means of correcting for instrument accommodation from automated refraction. Finally, inferences regarding other minority groups in other parts of China are of limited reliability, as the ethnic makeup in Yunnan differs from that in other areas of China. Nonetheless, the challenges faced by minority peoples in Yunnan, including limited access to education and healthcare, and the lessons from the current study on how these challenges might be overcome, are of potential relevance to other excluded and underserved groups in China and elsewhere.

Despite its limitations, this manuscript is among the first to give information not only on the prevalence of refractive error among minority children in a Chinese region with a significant minority population, but also importantly provides data on their baseline access to spectacles and willingness to accept services. As such, it should be of use to those planning programs of spectacles delivery in similar populations in China.

## Supporting information

S1 Table “B aseline student questionnaire in Chinese”. (PDF)
