# Supplementary material for: Population prevalence of myopia, glasses wear and free glasses acceptance among minority versus Han schoolchildren in China
Source: PLoS One. 2019 Apr 18;14(4):e0215660. doi: 10.1371/journal.pone.0215660 (PMC6472783; doi:10.1371/journal.pone.0215660)
Supplement: S1 Table — (PDF) [file pone.0215660.s001.pdf]

调查员编码：____________　　　　　　　　　　　　　　　填表日期：______________

问卷编码：□□□□□□□□□

小学生基本情况调查

学 生 问 卷

同学你好：

今天让大家来填写一份问卷。我们会为你的答案保密，你的答案不会告诉调查组以外的任何人。这些问题也并没有标准答案， 希望你不要有任何顾虑，跟随我们准确、如实、完整地填写这份调查表。如果你在填这份问卷时有任何问题，请随时举手问我们。衷心感谢你对本次调研工作的积极支持与配合！祝你身体健康！学习进步！

_________省_________县（区）___________镇（乡）__________小学___年级_____班

姓名：________________　性别：_______________　年龄：_____________

名族：（请在下表中你所在的名族后打钩）

汉族		傣族		彝族		回族		苗族	
白族		壮族		哈尼族		拉枯族		傈僳族	
瑶族		畲族		土家族		布朗族		景颇族	
德昂族		佤族		阿昌族		纳西族		其他，请注明	

答题须知：

1. 每个表格中有三列：“问题”、“单位/选项”和“答案”。如果是选择题，请将您认为最符合您情况和想法的选项的**代码**填在选项后面“答案”列的空格里。如果是问答题，请把您的答案用文字填在选项后面“答案”列的空格里。
2. 如无特别说明，本问卷中所有的问题都只选一个答案。
3. 数字要写阿拉伯数字，比如1、2、3。
4. 如果选择题选项中有“其他，请说明____”这样的字样，您也选了这项，请在“答案”列中填上这项的代码，并在“答案”列同时填上需要说明的内容。
5. **不能用铅笔**填写。

一、基本信息

问题	选项	答案
1.你家共有几口人？	人	
2.你有几个亲兄弟姐妹（指和你同父母的，没有就写“0”）？	个	哥哥： 姐姐：<br>弟弟： 妹妹：
3.如果**有亲兄弟姐妹**，有几个戴眼镜？	个	哥哥： 姐姐：<br>弟弟： 妹妹：
4.你每天是怎么上学的？	1=步行；<br>2=骑自行车；<br>3=坐校车；<br>4=坐公交车；<br>5=坐私家车；<br>6=其他，请注明	
5.你爸爸今年多大年纪？	周岁	
6.你爸爸戴眼镜吗？（**太阳镜**除外）	1=戴； 2=不戴	
7.你爸爸的文化程度？	1=没上过学；2=小学；<br>3=初中； 4=高中或中专；<br>5=大专； 6=大学及以上	
8.你爸爸在哪里工作？	1=本地工作（包括务农）；<br>2=县城打工；<br>3=在外地打工	
9.你妈妈今年多大年纪？	周岁	
10.你妈妈戴眼镜吗？（**太阳镜**除外）	1=戴； 2=不戴	
11.你妈妈的文化程度？	1=没上过学；2=小学；<br>3=初中； 4=高中或中专；<br>5=大专； 6=大学及以上	
12.你妈妈在哪里工作？	1=本地工作（包括务农）；<br>2=县城打工；<br>3=在外地打工	
13.你家的电话号码是多少？	可填写多个手机或座机号码，号码之间用逗号分开	

二、 座位情况

问题	选项	答案
14.你现在坐在教室的第几排？	排	
15.你现在坐在教室的第几列（从**你的左边**数起）？	列	

在下面的表格中，请填入对应的关系中你认为**关系最好**的五个**同班同学现在坐**的座位。

16.平时和你**一起玩**的同学：	第 1	第 2	第 3	第 4	第 5
（在这一排写下同学的名字）					
排					

列					
17.平时和你**一起学习**的同学：	第 1	第 2	第 3	第 4	第 5
（在这一排写下同学的名字）					
排					
列					
18.和你**住的最近**的同学：	第 1	第 2	第 3	第 4	第 5
（在这一排写下同学的名字）					
排					
列					

三、时间偏好

在以下四题中依次选出你心仪的给零花钱方式			选项
19.	A. 一个月以后 的 100 元	B. 四个月以后 的 100 元	
20.	A. 一个月以后 的 100 元	B. 四个月以后 的 120 元	
21.	A. 一个月以后 的 100 元	B. 四个月以后 的 140 元	
22.	A. 一个月以后 的 100 元	B. 四个月以后 的 160 元	
在以下四题中依次选出你心仪的给零花钱方式			选项
23.	A. 一个月以后 的 100 元	B. 六个月以后 的 100 元	
24.	A. 一个月以后 的 100 元	B. 六个月以后 的 150 元	
25.	A. 一个月以后 的 100 元	B. 六个月以后 的 200 元	
26.	A. 一个月以后 的 100 元	B. 六个月以后 的 250 元	
在以下四题中依次选出你心仪的给零花钱方式			选项
27.	A. 四个月以后 的 100 元	B. 七个月以后 的 100 元	
28.	A. 四个月以后 的 100 元	B. 七个月以后 的 120 元	
29.	A. 四个月以后 的 100 元	B. 七个月以后 的 140 元	
30.	A. 四个月以后 的 100 元	B. 七个月以后 的 160 元	

四、视力现状

问题	选项	答案
31.你每天放学后看书、写作业的时间？	1=不看书或写作业；<br>2=半小时以内；	

	3=半小时到一小时；<br>4＝一小时至两小时；<br>5=二小时至三小时；<br>6=三小时以上	
32.老师平时上课出的测试题，有多大比例是写在黑板上的？	1=全部； 2=大部分；<br>3=一半左右； 4＝很少；<br>5=没有	
33.你平时在座位上能不能看得清楚黑板上的字？	1=看得清楚； 2=看不清楚	
34.你觉得你近视吗？	1=是； 2=否	
35.自上学期以来，你参加过学校组织的视力检查吗？	1=是； 2=否	
36.你的检查结果？	1=没有参加； 2=视力正常；<br>3=近视； 4=不知道结果；<br>5=其他，请说明。	
37.自上学期以来，家里人是否带你检查过视力？	1=是； 2=否	
38.你的检查结果？	1=没有参加； 2=视力正常；<br>3=近视； 4=不知道结果；<br>5=其他，请说明。	
39.你觉得戴眼镜难看吗？	1=难看； 2=有点难看；<br>3=一般； 4=不难看	
40.你们班有同学因为戴眼镜被别的同学笑话吗？	1=有； 2=没有	
41.你们班有同学因为戴眼镜被别的同学欺负吗？	1=有； 2=没有	
42.你的好朋友当中，有人近视吗？	1=有； 2=没有；<br>3=不知道	
43.那你的好朋友当中，有人有眼镜吗？	1=有； 2=没有(跳到45题)；<br>3=不知道	
44.那他们平时戴眼镜吗？	1=基本不戴； 2=学习的时候戴；<br>3=经常戴	
45.你有眼镜吗？	1=有； 2=没有（**跳到第 54 题**）	
46.你戴眼镜多少年了？	年	
47.你配过几副眼镜？	副	
48.你平时戴眼镜吗？	1=基本不戴； 2=学习的时候戴；<br>3=经常戴（**跳到第 50 题**）	
49.如果你不是经常戴眼镜，为什么？<br>**（只能选一个答案）**	1=担心戴了眼镜后视力越来越差；<br>2=担心戴了眼镜被别人笑话；<br>3=不戴眼镜也勉强看得见，等以后看不清楚了再戴；<br>4=觉得戴着眼镜行动（或体育活动）不方便；<br>5=戴眼镜不舒服，会觉得头晕、鼻梁受压等；<br>6=其他，请注明	
50.你的家长对你戴眼镜是什么态度？	1=支持； 2=无所谓； 3=反对	

51.你最近一次配（换）眼镜是在哪一年？	年	
52.你的眼镜是在哪里配的？	1=医院；　　2=眼镜店；<br>3=其他，请说明	
53.你配眼镜花了多少钱？<br>**（本题回答结束跳到第 55 题）**	1=100 元以下；<br>2=100 元到 199 元；<br>3=200 元到 299 元；<br>4=300 元到 399 元；<br>5=400 元到 499 元；<br>6=500 元及以上	
54.你为什么没有配眼镜？	1=不近视<br>2=担心戴了眼镜后视力越来越差；<br>3=担心戴了眼镜被别人笑话；<br>4=现在勉强看得见，以后看不见了再配；<br>5=觉得戴着眼镜行动(或体育活动)不方便；<br>6=戴眼镜不舒服，会觉得头晕、鼻梁受压等；<br>7=眼镜比较贵；<br>8=家里人没时间带着去配；<br>9=其他，请注明	

五、眼镜使用问题 （对于以下说法，同意写 1；不同意写 2；不知道写 3）

55.眼保健操能矫正近视	1=同意；2 不同意；3=不知道	
56.戴眼镜能矫正近视	1=同意；2 不同意；3=不知道	
57.对于成绩**比较差**，同时又**近视**的学生，戴眼镜会**提高**学习成绩	1=同意；2 不同意；3=不知道	
58.对于成绩**比较差**，但**没有近视**的学生，戴眼镜会**提高**学习成绩	1=同意；2 不同意；3=不知道	
59.对于成绩**比较好**，同时又**近视**的学生，戴眼镜会**降低**学习成绩	1=同意；2 不同意；3=不知道	
60.对于成绩**比较好**，但**没有近视**的学生，戴眼镜会**降低**学习成绩	1=同意；2 不同意；3=不知道	
61.对于**看不清楚**黑板的学生，戴眼镜会让他们看的**更清楚**	1=同意；2 不同意；3=不知道	
62.对于**看的清楚**黑板的学生，戴眼镜会让他们看的**不清楚**	1=同意；2 不同意；3=不知道	
63.戴眼镜会导致视力越来越差	1=同意；2 不同意；3=不知道	
64.小学生戴眼镜是不是还太早	1=同意；2 不同意；3=不知道	

六、家庭资产拥有情况

资产类别	1=有；2=没有	资产类别	1=有；2=没有
65.小汽车		66.照相机	
67.货车		68.洗衣机	
69.摩托车或电动车		70.空调	
71.拖拉机		72.热水器	

73.大型农机具		74.煤气/液化气炉具	
75.电脑		76.抽油烟机	
77.拉网线		78.电冰箱或冰柜	
79.电视机		80.抽水马桶	
